# A Dataset of Electrical Components for Mesh Segmentation and Computational Geometry Research

**DOI:** 10.1038/s41597-024-03155-w

**Published:** 2024-03-22

**Authors:** Benedikt Scheffler, Patrick Bründl, Huong Giang Nguyen, Micha Stoidner, Jörg Franke

**Affiliations:** Friedrich-Alexander-Universität Erlangen-Nürnberg, Institute for Factory Automation and Production Systems (FAPS), Nuremberg, Germany

**Keywords:** Electrical and electronic engineering, Mechanical engineering, Scientific data

## Abstract

Data quality is of crucial importance in the field of automated or digitally assisted assembly. This paper presents a comprehensive data set of triangle meshes representing electrical and electronic components obtained by scraping Computer Aided Design (CAD) models from the Internet. Consisting of a total of 234 triangle meshes with labelled vertices, this data set was specifically created for segmentation tasks. Its versatility for multimodal tasks is underscored by the presence of various labels, including vertex labels, categories, and subcategories. This paper presents the data set and provides a thorough statistical analysis, including measures of shape, size, distribution, and inter-rater reliability. In addition, the paper suggests several approaches for using the data set, considering its multimodal characteristics. The data set and related findings presented in this paper are intended to encourage further research and advancement in the field of manufacturing automation, specifically spatial assembly.

## Background & Summary

**Fig. 1:**
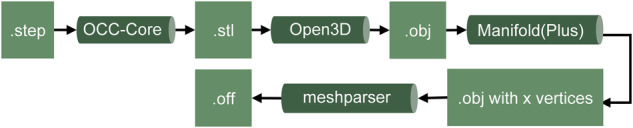
Conversion and preprocessing of aggregated STEP files.

Manufacturers are under increasing pressure to deliver quality products while meeting their customers’ unique requirements. Many companies also face the growing challenge of efficiently producing customized products in a cost-effective manner^1^. One approach for this can be the use of automation or digital assistance solutions. However, there is often a lack of high-quality data, which companies have had to reengineer at great expense, creating a significant barrier to deploying such solutions^2^. Key to this is the need for precise and consistent geometric representations that support the integration of Design for Testability methodologies^1^. With this in mind, the research presents an in-depth analysis of 3D triangle meshes, which are critical components in understanding the geometric intricacies of electronic components. The study design involved collecting labels for 24 triangle meshes from three different raters, resulting in a total of 72 label files. These labels underwent a rigorous statistical evaluation, employing inter-rater reliability metrics like Cohen’s kappa, Fleiss’ kappa, and Randolph’s kappa, as well as advanced geometric descriptors such as optimal transport distance (OTD) and heat kernel signature (HKS). Descriptive statistics were also leveraged to provide foundational insights. The overarching objectives of this study are twofold: To establish a dependable benchmark for human performance in 3D mesh segmentation, a critical step is taken to define the essential features for the automated assembly of electrical and electronic components in control cabinets. This benchmark aims to serve as an invaluable guide for the development of future automation algorithms. Second, the study rigorously evaluates the geometric similarity between multiple mesh resolutions with the intent of confirming their interchangeability in applications where the granularity of tessellation is not a deciding factor. The data set can be used for many different use cases. Besides the domain-related use case for learning the geometric features of electronic components, the data set is additionally suitable for benchmarking of most mesh algorithms. Concerning triangle mesh downsampling algorithms, this data set holds a pivotal role. Apart from the semantic segmentation, the instance segmentation of the features is also conceivable, as well as the determination of the features by means of other methods such as 3D bounding boxes. Figure 1 shows the data preprocessing pipeline to convert standard for the exchange of product model data (STEP) files into corresponding suitable formats for deep learning.

## Methods

### Aggregation

As part of the work, 46,068 STEP files of electrical components were scraped from the internet. These components stem from a total of 75 different manufacturers. In addition to the components themselves, the metadata fields shown in Table 1 were scraped.

**Table 1:** Metadata scraped for each component in the data set.

Metadata Fields		
Name	Category	Length [mm]
Manufacturer	Subcategory	Current [A]
Part Number	Series	Article Status
Component Type	Width [mm]	Component Description
Technology	Height [mm]	External Document

**Fig. 2:**
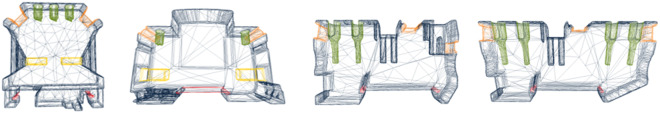
Example labelled triangle meshes in the data set.

The completeness of the properties for all components is not given, because not all manufacturers list all mentioned information, which additionally shows the need for software-based information generation. The property “External document” refers to the official manufacturer documentation of the component. Since many components differ only in electrical characteristics but have identical geometry, all files were hashed using a Secure Hash Algorithm (SHA) 256 algorithm to identify geometric duplicates. This resulted in 21,134 geometrically different STEP files. During data preprocessing, a STEP file is converted into a STL triangle mesh via Open Cascade Technology. Triangle meshes and point clouds can be represented much better as tensors than STEP files and are therefore more suitable for Deep Learning (DL). Previous approaches of DL based on triangle meshes mostly work starting from Wavefront OBJ (OBJ) or object file format (OFF) files. Accordingly, the goal of data preprocessing is to convert and scale the files efficiently and correctly. All files in the data set can be filtered using the technology, category and subcategory to work with a subset. User-specific filters based on the scraped metadata can also be used to select components of a certain size, manufacturer, or electrical characteristics. The data set was then filtered to include only components relevant to control cabinet assembly, leaving 4,729 components. Figure 2 shows sample triangle meshes from the data set with their visualized geometric features.

### Annotation

**Fig. 3:**
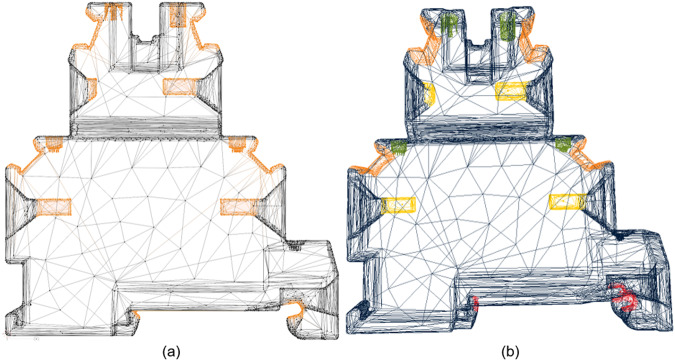
Labelled vertices in Blender (**a**) and exported labels for each class visualized via Open3D (**b**).

After filtering the files, a manual inspection of all remaining files was performed. The remaining files were converted to OBJ files, 234 of these files were subsequently labelled in Blender into five different classes as shown in Fig. 3: housing (dark blue, class ‘0’), contacting (green, class ‘1’), snap-on point (red, class ‘2’), cable entry (yellow, class ‘3’) and labelling area (orange, class ‘4’). The classes were chosen following extensive discussions with three domain experts from the control cabinet industry. Through thorough considerations on the final application and domain-specific use cases, the selected classes were determined based on their relevance to the assembly and disassembly processes of control cabinets. The labelling was done vertex-wise and all vertices were assigned to exactly one vertex group representing the different classes.

This chapter considers only the remaining 234 files. Special attention was paid to their geometry when selecting the labelled components. Due to the similarity and only minimal differences of some components, some criteria were considered during the selection of the components to prevent overfitting to one component shape. Only components that met one of the following criteria were selected:

- Components with differences in width, height or length
- Components with different numbers of contacts, snap-on points, cable entries or labelling areas
- Components with features that deviate from the standard, e.g. angled contacts
- Components with strongly asymmetrical features

Taking these criteria into account, a diligent effort was made to mitigate potential data bias as much as possible by having the test data labelled by three independent people. The majority vote was then calculated and used as ground truth. By combining the annotations of three different people, human-level performance can be approximated. This also provides valuable insight into the extent to which the data is consistently labelled by experts and how the labels affect the final segmented features.

### Post processing

Since errors can occur during the conversion, they were corrected first and only then the scaling was performed. The goal is to generate a robust 2-manifold. The conversion in this work is done using an octree to represent the original mesh and constructs the surface by isosurface extraction^3^. Finally, a projection of the vertices onto the original mesh is performed to achieve high precision. In addition, a watertight 2-manifold is recovered by extracting outer surfaces between occupied and empty voxels and a projection-based optimization method^4^.

### Labelling format

**Fig. 4:**
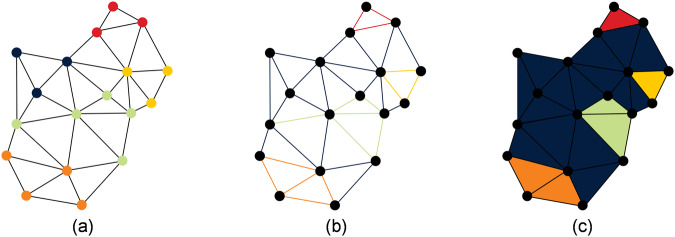
Exemplary difference between vertex labels (**a**), edge labels (**b**) and triangle labels (**c**).

The files are available as high-resolution STL and downsampled OBJ and OFF files. While OBJ files represent exclusively a format for the representation of triangle networks, polygon networks can be represented additionally by means of OFF files. However, the double submission of the files is exclusively due to the fact that already published and established neural networks like MeshCNN^5^ or DiffusionNet^6^ mostly accept one of these two files as input in their inference pipeline. This allows for quick and easy experimentation with many different network architectures on the data set. For triangle meshes, vertex-wise, edge-wise or triangle-wise labels are common. The conversion of the labels into one of the other two formats is easily possible under definition of uniform conditions. In the context of the work, the conversion of the labels into other labels was also provided. The difference between vertex, edges and triangle labels is exemplified in Fig. 4.

It can be seen that the conversion is possible without any problems in the direction shown from (a) to (c), but it is not lossless. A conversion back from (c) to (a) would, for example, label the leftmost green vertex blue instead of green. Since both STL and OBJ and OFF files number the vertices and triangles sequentially from the beginning to the end of the file, label assignment is easy for vertex or triangle labels. In this case, the labels consist of a one-dimensional tensor of shape *n*×1 with *n* equal to the number of vertices or triangles of the triangle mesh depending which labelling format is chosen. In the case of edge labels, an implementation following Hanocka *et al*. is used.

## Data Records

The Electrical and Electronic Components Dataset^7^ is available at the Harvard Dataverse. Components are labelled vertex-wise. The labels are in a txt-format and represent a one-dimensional tensor. In addition, components are labelled for classification tasks in technology, category, and subcategory. Possible values for these are the following classes:

- Technology: Cabinet Engineering; Not Categorized; Fluid Engineering; Electrical Installation; Electrical Engineering
- Category: Cables and Condcutors; Measuring + reporting devices; KNX (EIB); Power Source; Loads; Switch + Protection; Contactor, Relays; Plugs; Terminals
- Subcategory: Accessories

**Fig. 5:**
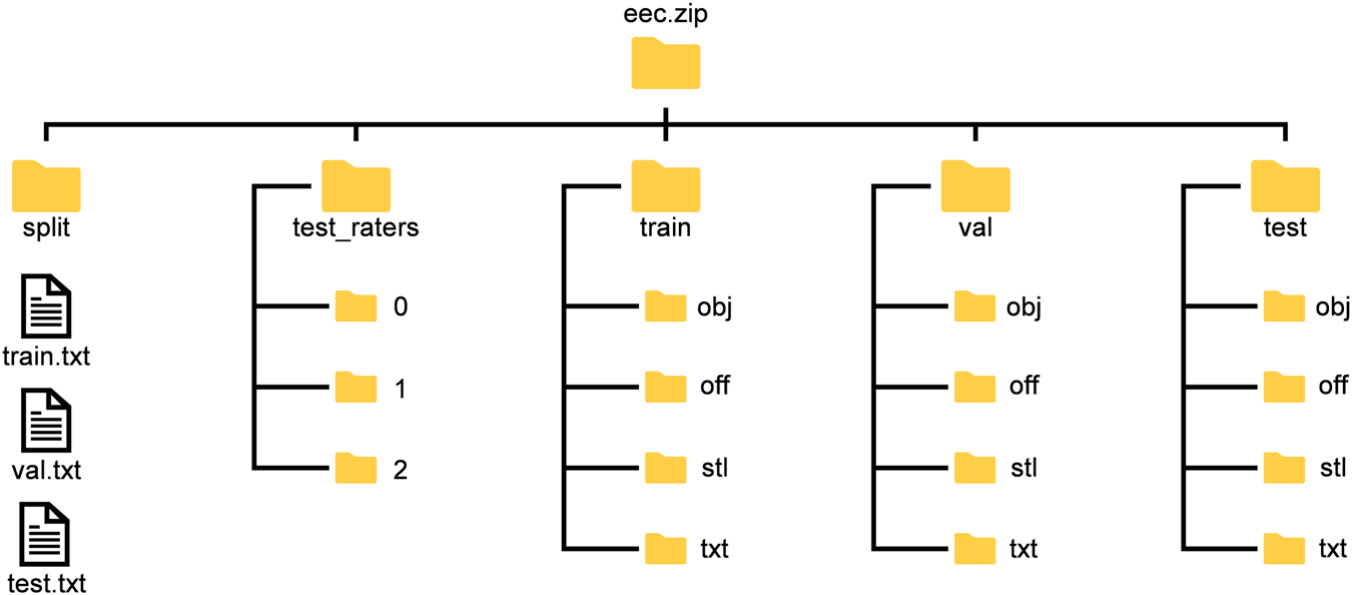
Folder structure of the Electrical and Electronic Components Dataset.

All triangle meshes are scaled to about 12,000 triangles and are available as OBJ. In addition, the triangle meshes are in multiple form. The Electrical and Electronic Components Dataset consists of five subfolders as shown in Fig. 5.

In the “split” folder, the three files “train.txt”, “val.txt” and “test.txt” each contain the names of the components that are intended as a train/val/test split. The subfolder “test_raters” contains the test labels of the three independent labellers. All three subfolders contain 24 files. The “train”, “val”, and “test” folders are identical in structure and contain the triangle meshes in OBJ, OFF, and STL formats, respectively. While the former both are identical, the STL files represent triangle meshes with a higher resolution. The choice between OBJ and OFF files is based solely on the final algorithm used. The labels in the “test” folder were not manually relabelled but represent the majority vote of the other three labellers.

## Technical Validation

In the field of data-driven research, the quality of a data set is of utmost importance, especially when it is based on manual annotations. This technical validation aims to provide a robust evaluation of a data set consisting of labelled triangle meshes. Four critical aspects are addressed: internal consistency, inter-rater reliability, geometric mesh similarity, and feature similarity.

### Internal consistency

The consistency of labelling, particularly focusing on the mode and median being uniformly zero across all raters and data set partitions, underscores a coherent labelling strategy. This cohesiveness in central labels provides a stable and reliable baseline for subsequent vertex-wise analyses in geometric deep learning and other related applications. Ensuring such uniformity across different segments signifies that the labelling process was methodologically sound and adhered to a consistent norm or reference, thereby enhancing the reliability and applicability of the data set. A meticulous examination of the spread and variability in the labels, evidenced by subtle fluctuations in standard deviation values ranging from 1.1581 (validation set) to 1.2239 (rater 0), provides an implicit assurance of the data set’s integrity. This subtle range in spread does not allude to erratic variability but rather indicates a controlled and consistent labelling process. Furthermore, the tight range of variance values also corroborates the data set’s stability, providing a controlled environment where the data complexity is maintained without introducing unintended biases or skews. The data’s kurtosis and skewness were reviewed to ensure that any variability and asymmetry in the distribution were authentic and not a result of inconsistencies or errors in the labelling process. The validation set, exhibiting a pronounced kurtosis of 2.813, was specifically scrutinized to validate that it genuinely contains more extreme or rare cases, thereby enhancing the robustness of the data set by encompassing a wide array of potential real-world scenarios. The hierarchical pattern observed in the frequency distribution of each label, with label ‘0’ considerably dwarfing the others and comprising about 68% to 73% of the data set, was meticulously validated to ensure it reflects genuine data characteristics. The subsequent labels (‘1’ through ‘4’) present in a diminishing frequency order, each serving a specific and consistent role in representing various complexities and nuances in the triangle meshes. This hierarchical and consistent presence of labels, ensuring that neither is neglected nor disproportionately represented, validates that the data set can faithfully represent and be applied to a variety of network complexities and challenges.

### Inter-rater reliability

Technical validation is crucial for establishing the reliability and applicability of this triangle mesh data set, both in academic and industrial contexts. The validation process begins with a thorough analysis of inter-rater reliability, employing multiple metrics such as Cohen’s, Fleiss’, and Randolph’s kappa.

These metrics, with all kappa statistics above 0.81, indicating almost perfect agreement levels^8^, are further supported by t-tests on continuous performance measures. This multi-metric approach robustly validates the reliability of the data set, making it useful for a range of applications, including high-performance machine learning models. In particular, the value of the data set is tied to its manually annotated labels, the reliability of which is assessed using a variety of metrics. While percentage agreement provides a basic measure, it lacks the nuance provided by the kappa statistic *κ*, which accounts for random agreement. This multi-faceted evaluation ensures the reliability of the data set, especially considering that these annotations serve as ground truth for future analyses.

1 $$\kappa =\frac{{P}_{o}-{P}_{e}}{1-{P}_{e}}$$

2 $${P}_{o}=\frac{{\sum }_{i=1}^{k}{n}_{ii}}{N},with\,N=\mathop{\sum }\limits_{i=1}^{k}\mathop{\sum }\limits_{j=1}^{k}{n}_{ij}$$

3 $${P}_{e}=\mathop{\sum }\limits_{i=1}^{n}\left({P}_{1i}\times {P}_{2i}\right)$$

Cohen’s kappa not only quantifies the degree to which raters agree but also adjusts for what could be expected by random chance. In case of Cohen’s kappa, the observed agreement *P*_*o*_ is given by the ratio of the number of vertices on which both raters agree to the total number of vertices rated, where *k* is the number of categories and *N* is the total number of vertices. The expected agreement *P*_*e*_ of two raters is defined as the probability that rater one picks class *i* times the probability that rater two picks class *i*. In our data set, Cohen’s kappa values for the corresponding rater pairs are 0.9225 for rater (0,1), 0.8388 rater (0,2) and rater 0.8561 (1,2). This suggests “substantial” agreement based on Landis and Koch’s scale^9^. However, Cohen’s kappa is best suited for pairwise comparisons and may not fully capture the dynamics in scenarios with more than two raters, even when averaging the values^10^. Therefore the significance of Fleiss’ kappa^11^ becomes evident. Designed as a statistical measure for assessing the reliability of agreement between a fixed number of raters when assigning categorical ratings, Fleiss’ kappa extends the utility of Cohen’s kappa to multi-rater scenarios^10^. It is defined as^11^

4 $${\kappa }_{f}=\frac{\bar{P}-\overline{{P}_{e}}}{1-\overline{{P}_{e}}}$$

5 $$\bar{P}=\frac{1}{N}\mathop{\sum }\limits_{j=1}^{N}\left(\frac{1}{n\left(n-1\right)}\mathop{\sum }\limits_{k=1}^{K}{n}_{jk}\left({n}_{jk}-1\right)\right)$$

6 $$\overline{{P}_{e}}=\mathop{\sum }\limits_{k=1}^{K}{\left(\frac{1}{N\times n}\mathop{\sum }\limits_{j=1}^{N}{n}_{jk}\right)}^{2}$$

where $\bar{P}$ and $\overline{{P}_{e}}$ is the average observed and expected agreement among all raters respectively. Here *n*_*jk*_ is the number of raters who assigned the *k*^*th*^ category to the *j*^*th*^ subject with *n* being the number of raters, *K* the number of categories and *N* the number of subjects^11^. The calculated Fleiss’ kappa value is 0.8561, corroborating the finding of substantial agreement among the raters. While kappa statistics are widely used, they are not without limitations. They can be sensitive to trait prevalence and base rates, potentially leading to paradoxical or misleading results. To mitigate these limitations, we also employed Gwet’s AC1^12^ which is defined by:

7 $$AC1=\frac{{P}_{o}-{P}_{c}}{1-{P}_{c}}$$

8 $${P}_{o}=\frac{1}{N}\mathop{\sum }\limits_{i=1}^{k}{X}_{ii}$$

9 $${P}_{o}=\frac{1}{{N}^{2}}\mathop{\sum }\limits_{i=1}^{k}{\left(\mathop{\sum }\limits_{j=1}^{n}{X}_{ji}\right)}^{2}$$

*P*_*o*_ is the observed agreement among the raters and *P*_*c*_ is the agreement by chance^12^. Where *N* is the total number of meshes being rated and *X*_*ii*_ is the number of raters that agree on class *i* for a given vertex. *X*_*ij*_ is the number of raters that assigned the *j*^*th*^ vertex to class *i*. With a value of 0.9272, it provides an additional layer of confirmation for the substantial agreement among raters. The convergence of these different metrics while being largely consistent lends robustness to the reliability of the data set. Similarly, the independent hypothesis tests from Table 3 ensure that the data set is not only internally consistent, but also a reliable tool for a variety of external applications, ranging from academic research, computational geometry research to machine learning models aimed at approximating human-level performance in mesh segmentation.

In addition, the Brennan-Prediger kappa^13^ (0.9203), Conger’s kappa^14^ (0.87218), and Krippendorff’s alpha^15^ (0.87215) were also evaluated, confirming the high agreement.

### Geometric similarity

Understanding the geometric similarity between different mesh formats – in particular OFF and STL – is essential not only for computational analysis, but also for ensuring that geometric information is not lost during downsampling. This study uses the Hausdorff distance, HKS, and p-Wasserstein distance to effectively tackle shape representation and comparison. The Hausdorff distance offers a precise measure for maximum discrepancies, vital for exact shape matching. HKS provides deep insights into local geometric features, enhancing our ability to distinguish between similar shapes through isometric invariance. The p-Wasserstein distance complements these by quantifying distribution transformations based on spatial geometry, ensuring a comprehensive analysis. The combination of these three descriptors ensures that the similarity measurement is both thorough and efficient. The Hausdorff distance^16^ lends itself to quantifying the extent to which two subsets of a metric space are close defined by^16^:

10 $$\begin{array}{ccc}H(A,B) & = & \max (h(A,B),h(B,A))\\ h(A,B) & = & \mathop{\max }\limits_{a\in A}\,\mathop{\min }\limits_{b\in B}\Vert a-b\Vert \end{array}$$

Surprisingly, a high Hausdorff distance was found between the OFF and STL formats, with a mean average distance of 14.1604 mm. This is significant because the distance is measured in the same unit as the meshes themselves, which is millimeters. While this could be a warning signal in most contexts, it is important to remember that the Hausdorff distance is very sensitive to mesh tessellation. Considering that the OFF and STL formats have different tessellation granularity, the high Hausdorff distance is more of a warning sign than a judgment of geometric dissimilarity. This therefore highlights the need for a multimetric approach. To further investigate the surprisingly high Hausdorff distance, the optimal transport distance^17^ in the form of p-Wasserstein distance^18^ and heat kernel signature^19^ were measured as more nuanced shape descriptors. The first also known as the Wasserstein-Kantorovich-Rubinstein^18^ distance or in the context of computer vision referred to as Earth Mover’s^18,20^ distance provides a robust way to measure shape similarity by considering the cost of transforming one shape into another^18^:

11 $${d}_{{\mathcal{W}},p}^{Z}({\mu }_{A},{\mu }_{B})\,:=\mathop{{\rm{\inf }}}\limits_{\mu \in {\mathcal{M}}({\mu }_{A},{\mu }_{B})}{\left(\mathop{\int }\limits_{A\times B}{d}^{p}(a,b)d\mu (a,b)\right)}^{1/p}$$

On the other hand, HKS as a function of time t, offers a spectral analysis of the shape, encapsulating local and global geometric properties. The HKS^19^ is defined as the diagonal (*x* = *y*) of the heat kernel *k*_*t*_ with *ϕ* being the eigenfunctions of the Laplace-Beltrami-Operator evaluated at *x* and y respectively and *λ*_*i*_ being the corresponding eigenvalues^21^:

12 $${k}_{t}\left(x,y\right)=\mathop{\sum }\limits_{i=0}^{\infty }{e}^{-{\lambda }_{i}t}{\phi }_{i}\left(x\right){\phi }_{i}\left(y\right)$$

13 $$HKS\left(x,t\right)={k}_{t}\left(x,x\right)=\mathop{\sum }\limits_{i=0}^{\infty }{e}^{-{\lambda }_{i}t}{\phi }_{i}{\left(x\right)}^{2}$$

Both the OFF and STL mesh formats exhibit a remarkable degree of geometric congruence, as substantiated by the HKS and OTD. The HKS values were computed on the normalized meshes, using 16 eigenvalues^6^ for computational efficiency, and further analyzed through dynamic time warping^22^. This analysis yielded a mean HKS value of 3.5677 × 10^−8^ and a maximum of 5.36 × 10^−7^, both extremely close to zero. These low HKS values strongly suggest that the diffusion processes on both mesh formats are nearly identical, affirming their intrinsic geometric similarity. Complementing this, the mean Earth Mover’s distance between the OFF and STL formats was found to be 9.8169, with a maximum distance of 148.1478. This metric, which quantifies the minimal “cost” of transforming one shape into another, further corroborates the geometric congruence indicated by the HKS values. Despite differences in tessellation granularity, these metrics collectively demonstrate that the OFF and STL formats are highly similar in their underlying geometry and can be considered interchangeable for applications where tessellation granularity is not a critical factor.

### Feature similarity

The detailed evaluation of feature similarity across different raters constitutes a vital component of the technical validation process in this study. This is of particular importance given the study’s overarching aim of facilitating the automated assembly of electrical and electronic components in control cabinets. For this purpose, distinct features were extracted from the main triangle meshes using the labels provided by each rater. These isolated, disconnected submeshes were then subjected to an exhaustive geometric analysis, which included computations of their surface area, the volume encapsulated by their convex hulls, and various centroids. Despite slight variations in labelling metrics like accuracy, precision, recall F1-score, and the Jaccard index among the raters, these differences had a negligible impact on the geometric attributes of the segmented features. The surface area of the labelled classes exhibited only small percentage differences as shown in Table 4.

The percentage of the total surface area occupied by all features shows minimal variation when compared to the surface area of the triangle meshes as a whole. Notably, certain classes, such as 2 and 3, occupy a significant amount of surface area, although the vertex label frequencies in Table 2 suggest otherwise. This indicates that classes 2 and 3 may require fewer vertices for representation, indicating simpler geometry. Consequently, it would be prudent to consider either a surface-based segmentation approach or one based on shape descriptors to address the label imbalance.

**Table 2:** Data consistency checks of the labels of the three independent raters, the train, validation and test split and the total data set.

	Rater 0	Rater 1	Rater 2	Train	Val	Test	Total
Mode	0	0	0	0	0	0	0
Median	0	0	0	0	0	0	0
Std	1.2239	1.2089	1.1953	1.1858	1.1581	1.2155	1.1837
Variance	1.4979	1.4614	1.4289	1.4061	1.3412	1.4775	1.4012
Minimum	0	0	0	0	0	0	0
Maximum	4	4	4	4	4	4	4
Kurtosis	1.7803	1.7448	1.4508	2.1561	2.8135	1.7424	2.2306
Skewness	1.7709	1.7547	1.6580	1.8585	2.0247	1.7566	1.8792
Frequency 0	0.6898	0.6897	0.6782	0.7017	0.7345	0.6903	0.7071
Frequency 1	0.1494	0.1465	0.1418	0.1471	0.1264	0.1456	0.1428
Frequency 2	0.0285	0.0321	0.0489	0.0282	0.0285	0.0323	0.0287
Frequency 3	0.0612	0.0674	0.0762	0.0596	0.0479	0.0649	0.0578
Frequency 4	0.0712	0.0643	0.0549	0.0634	0.0628	0.0669	0.0636

**Table 3:** Validation of label quality using t-tests on the inter-rater accuracy, precision, recall, f1-score and Jaccard-Index.

	(0,1)		(0,2)		(1,2)	
	t-stat	p-value	t-stat	p-value	t-stat	p-value
Accuracy	−1.7151	0.0931	3.4263	0.0013	4.6360	2.95 × 10^−5^
Precision	−0.6240	0.5357	3.9274	0.0003	5.6648	9.18 × 10^−7^
Recall	−2.8747	0.9095	4.6117	0.0061	0.3678	3.19 × 10^−5^
F1-score	−1.7658	0.0841	3.3739	0.0015	6.2136	1.38 × 10^−7^
Jaccard-Index	−2.0423	0.0469	4.3256	0.0001	6.9027	1.28×10^−8^

**Table 4:** Average percentage area of each feature to the total area of the triangle meshes.

Rater	Class 1 (%)	Class 2 (%)	Class 3 (%)	Class 4 (%)
#0	2.0283	3.1186	3.4101	1.7505
#1	1.9647	2.1489	3.5954	1.5200
#2	1.9101	5.0279	4.3314	1.3522

## Usage Notes

The Electrical and Electronic Components Dataset^7^ is freely available at the Harvard Dataverse at 10.7910/DVN/D3ODGT. Throughout the software development, only open-source software was used. Blender and associated Python packages were used for labelling and exporting the labels. All statistical results can be replicated using the code in https://github.com/bensch98/eec-analysis. The script stats.py calculates all statistics in pandas DataFrames and exports them to CSV files. The implementation of the HKS is based on Sharp *et al*.

### Experimental application

The data set was already used in segmentation related tasks using MeshCNN and DiffusionNet^23^. In this proposed concept meaningful results were achieved across all segmentation classes based on the 234 models included in this data set. The results obtained enable the precise determination of feature vectors. This is particularly relevant for direction-dependent assembly and disassembly steps such as cable contacting. Due to the dispersed nature of information sources relevant to assembly of control cabinets, the generated directional vectors are of particular significance as a unified database.

### Existing data sets

Other noteworthy data sets that can be used for similar deep learning-based approaches from a purely technological perspective include the ABC^24^, SHREC^25^ or MPI FAUST^26^ data set. While SHREC and MPI FAUST have no domain reference and therefore tend to be unsuitable for the intended use case of assembly and disassembly of control cabinets, our Electrical and Electronic Components data set is a useful addition to the widely used ABC data set. A combination of both data sets in the form of training a more general foundation model based on the ABC data set and a fine-tuning using the data set presented by us offers additional potential alongside the proof of concept already presented by Bründl *et al*.^23^.

## Data Availability

Figures 2, 3 were generated using the data provided in the data set using the open source library Open3D^27^. To facilitate the reproduction of these figures, a copy of the raw data is included in the repository’s corresponding directory. Data set preparation was done with a combination of libraries, including Open3D^27^, numpy^28^ and bpy. Blender version 3.3.0 was used to label the 234 triangle meshes. Further on, various functions were programmed to interact with the data set. These include functions for scaling labels up and down, cropping triangle meshes by labels, converting vertex labels to triangle or edge labels and calculating surface, volume and cluster centroids of the cropped out meshes like the labelled regions. Additionally, based on the technology, category, and subcategory, the components can be filtered. All the software used in the study are open source available at https://github.com/bensch98/eec-analysis.
